# The Robson classification for caesarean section—A proposed method based on routinely collected health data

**DOI:** 10.1371/journal.pone.0242736

**Published:** 2020-11-30

**Authors:** Karen Triep, Nenad Torbica, Luigi Raio, Daniel Surbek, Olga Endrich

**Affiliations:** 1 Medical Directorate, Inselspital, University Hospital of Bern, Berne, Switzerland; 2 Department of Obstetrics and Gynecology, University Hospital of Bern, Berne, Switzerland; 3 Insel Data Science Center IDSC, Inselspital, University Hospital of Bern, Berne, Switzerland; Univesity of Iowa, UNITED STATES

## Abstract

**Background:**

With an increasing rate of caesarean sections as well as rising numbers of multiple pregnancies, valid classifications for benchmarking are needed. The Robson classification provides a method to group cases with caesarean section in order to assess differences in outcome across regions and sites. In this study we set up a novel method of classification by using routinely collected health data. We hypothesize i that routinely collected health data can be used to apply complex medical classifications and ii that the Robson classification is capable of classifying mothers and their corresponding newborn into meaningful groups with regard to outcome.

**Methods and findings:**

The study was conducted at the coding department and the department of obstetrics and gynecology Inselspital, University Hospital of Bern, Switzerland. The study population contained inpatient cases from 2014 until 2017. Administrative and health data were extracted from the Data Warehouse. Cases were classified by a Structured Query Language code according to the Robson criteria using data from the administrative system, the electronic health record and from the laboratory system. An automated query to classify the cases according to Robson could be implemented and successfully validated. A linkage of the mother’s class to the corresponding newborn could be established. The distribution of clinical indicators was described. It could be shown that the Robson classes are associated to outcome parameters and case related costs.

**Conclusions:**

With this study it could be demonstrated, that a complex query on routinely collected health data would serve for medical classification and monitoring of quality and outcome. Risk-stratification might be conducted using this data set and should be the next step in order to evaluate the Robson criteria and outcome. This study will enhance the discussion to adopt an automated classification on routinely collected health data for quality assurance purposes.

## 1 Introduction

**Fig 1:**
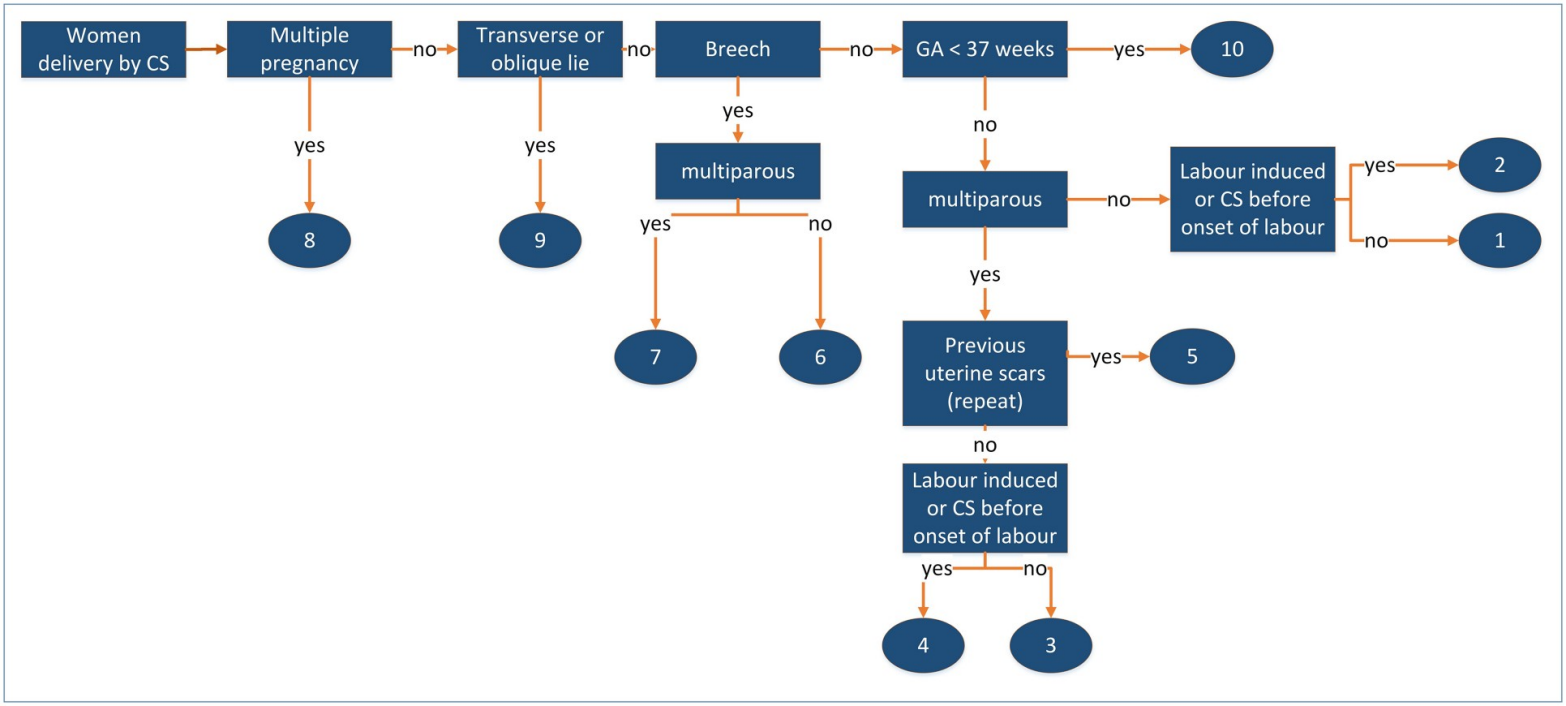
Flow chart Robson classification (Robson et al., 2002) according to WHO (2017) Robson classification: Implementation manual.

The caesarean section (CS) rate has been increasing during the last decades and the rate of CS varies among hospitals [1–3]. In Switzerland CS rates reach 33% and more. As many different classifications exist, the heterogeneity prevents valid comparisons between countries and hospitals [4–7]. A lack of clarity regarding indication and relevant obstetric history can be observed [8–10]. A commonly accepted classification of CS and its indications would allow an evaluation and comparison of the contributors to the CS rate and would make comparison between hospitals, regions, and countries possible [11]. The Robson classification of CS shows the CS rates in specific groups, see Fig 1 and S1 File, to help identifying possible reasons for this variation [12,13]. The Robson classification is recommended by the World Health Organization (WHO) [2,12,14]. It is based on pre labour, intrapartum and postpartum data. Currently it is not in use in Switzerland although it has been highly recommended [11].

As multiple births as well as elective CS numbers are rising in Switzerland and worldwide, the necessity of a valid benchmark of comparable groups, meaningful data and outcome measures becomes evident [5,6,9,15,16]. Meta analyses support the interest in the Robson classification [5,12].

The possibility to derive information from data is developing very fast, as routinely collected administrative and health data have accumulated during the last years and recent technology grants a higher degree of accessibility [17,18].

The Insel Gruppe Berne with approximately 62000 inpatient stays and more than 2200 annual births at the department of obstetrics and gynecology serves as a tertiary care center being obliged to treat high-risk patients (multiple pregnancies, preterm deliveries and repeat CS) [19].

To overcome different national specifications in statistics and lacking classifications for valid benchmark we conducted this study by evaluating different methods of classification and grouping with regard to costs and outcome parameters [16,18,20–26]. The technical approach offered the opportunity to develop a proof-of-concept for a Structured Query Language (SQL) query based medical classification process using routinely collected health data.

The novel approaches of this study are: i by using the technical capabilities and the amount of data from the clinical data warehouse extracting data with a minimum of requests (this study provides one of the first complex queries to the data), ii evaluating a medical classification by routinely collected health data and iii searching missing information by a novel text mining tool, which was tested (searching the electronic health record) and iv mapping the mother’s Robson class to the corresponding newborn. The study was conducted to elaborate a proof of concept of a complex medical classification which is based on routinely collected health data and which can be applied automatically. Moreover, it should be demonstrated that outcome related classes can be used for a standardized benchmarking.

We hypothesize i that routinely collected health data can be used to apply complex medical classifications and ii that the Robson classification is highly capable of classifying mothers and their corresponding newborn into meaningful groups with regard to outcome.

## 2 Methods

The study was conducted at the coding department and the department of obstetrics and gynecology Inselspital, University Hospital of Berne, Switzerland. The clinical data warehouse at the Inselspital contains administrative and medical data of all patients from the department of obstetrics and gynecology and the neonatology division. The data include the diagnoses codes (International Statistical Classification of Diseases and Related Health Problems 10th version, ICD) and procedure codes (Swiss classification of procedures, “CHOP”) of inpatient cases [27,28] and clinical data as the APGAR values (outcome related score to assess the neonate’s status) or laboratory results [10,25,29].

Inclusion criteria: Inpatient cases at the Inselspital Berne, discharges from 2014–2017 (224’331); all cases with a procedure code for caesarean section as procedure encoded (2’700), see S1 Fig. Filtering of the datasets was performed to make sure, that only classifiable and classified individuals remained in the data. The presence of a ‘null’–class of a relevant value, applied to individuals without the required information for classification, led to a removal of 3 entries of mother cases. After extracting the inpatient cases with CS, the corresponding newborns were mapped by a linkage code using the case identity number. Thus, it was possible to assign the mother’s Robson class to the corresponding neonate(s). Stillborn cases were excluded, as their diagnoses are not coded due to Swiss coding regulations. After data extraction and before analysis, the cases were anonymized (2’697 mother cases, 3’086 newborn cases), (total of cases see S1 Table).

Data were extracted from the clinical data warehouse and mapped (see S2 Fig). Outcome variables (referring to the clinical situation) were defined referring to literature (distinct ICD codes, intensive care treatment, ventilation hours, transfusion, 5-minute-APGAR score, Base Excess and pH value). An algorithm (SQL query) to apply the Robson classes using claims data (e.g. procedure codes, ICD codes, Diagnosis related groups (DRGs) [30], costs [31,32]) and otherwise routinely collected health data (laboratory, text, APGAR scores) was set up. The case related cost data were obtained from the REKOLE^®^- based cost-unit accounts [32], according to national standard. SwissDRG DRG type (related to length of stay, current Swiss inpatient reimbursement system of diagnosis related groups) and case related costs were used as surrogates of economic outcome variables in addition to the clinical outcome variables. The method of outlier calculation was executed according to the SwissDRG’s annually revised standard (batch grouper) [30]. The Robson classes were applied to both mother and child. Descriptive statistics for log10 case related costs and several clinical indicators (defined by e.g. ICD code, intensive care, ventilation, interventions/procedures) was conducted (S2 Table). The algorithm to propose Robson classes to the mothers’ cases was programmed in Transact-SQL, querying the necessary patient and case information and creating entries which represent the Robson class.

The Ethics Committee of the Canton Bern approved the study (KEK-Nr. Req-2017-00927) for quality assurance purpose. No informed consent was necessary. According to the regulations of the Bernese Ethics Committee no data combining a set of diagnoses and laboratory values on patient level (potentially identifying or sensitive patient information) can be labeled as fully anonymized. Supporting data, which can be given unrestricted access to, can be found in the supporting information file (contact information: https://www.gef.be.ch/gef/de/index/direktion/organisation/kek/kontakt.html). Data sharing is partly restricted as the original dataset contains de-identifying sets of coded diagnoses on patient level. Further data requests can be send to Dominique Furrer (dominique.furrer@insel.ch), local data protection manager of the institutional data access of the Insel Data Science Center, University Hospital of Bern, Berne, CH.

## 3 Results

The Robson classification demonstrated to be a highly usable method to aggregate relevant obstetric information corresponding to clinical indicators for both mother and child. The manual revision of 100 cases showed a high validity of the method, see Table 1.

**Table 1:** Validation of a random sample of 100 cases.

		Coding and/or discharge documentation positive	Coding and/or discharge documentation negative	total
SQL classification	first validation	98	2	100
	validation after correction of the code	100	0	100

The method itself successfully produced an application of the Robson criteria to the data of cases with CS (see Table 2).

**Table 2:** Count cases per Robson class and percent, cases with CS, per year.

	number of mother cases			
Robson Class	2014	2015	2016	2017
1	111	114	107	143
2	37	44	65	22
3	45	52	38	64
4	9	12	10	8
5	89	110	123	132
6	56	37	37	45
7	32	33	19	35
8	87	106	105	83
9	28	30	25	34
10	153	142	131	145
all	647	680	660	711
	percent of cases CS			
Robson Class	2014	2015	2016	2017
1	17.16	16.76	16.21	20.11
2	5.72	6.47	9.85	3.09
3	6.96	7.65	5.76	9.00
4	1.39	1.76	1.52	1.13
5	13.76	16.18	18.64	18.57
6	8.66	5.44	5.61	6.33
7	4.95	4.85	2.88	4.92
8	13.45	15.59	15.91	11.67
9	4.33	4.41	3.79	4.78
10	23.65	20.88	19.85	20.39
all	100	100	100	100

**Fig 2:**
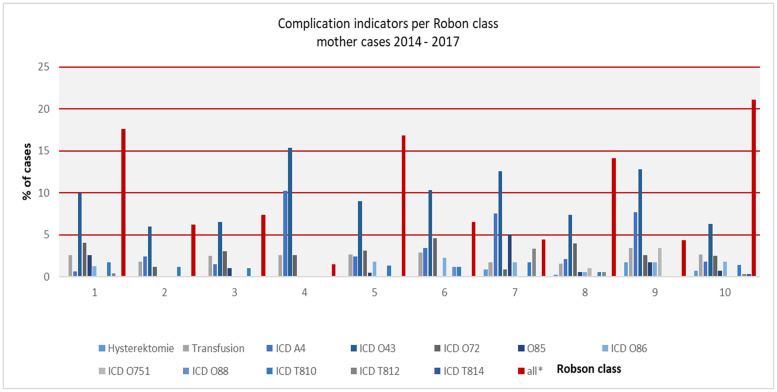
Complication indicators* per Robson class, % of cases, mother cases, 2014–2017. *ICD codes text see S2 Table.

**Fig 3:**
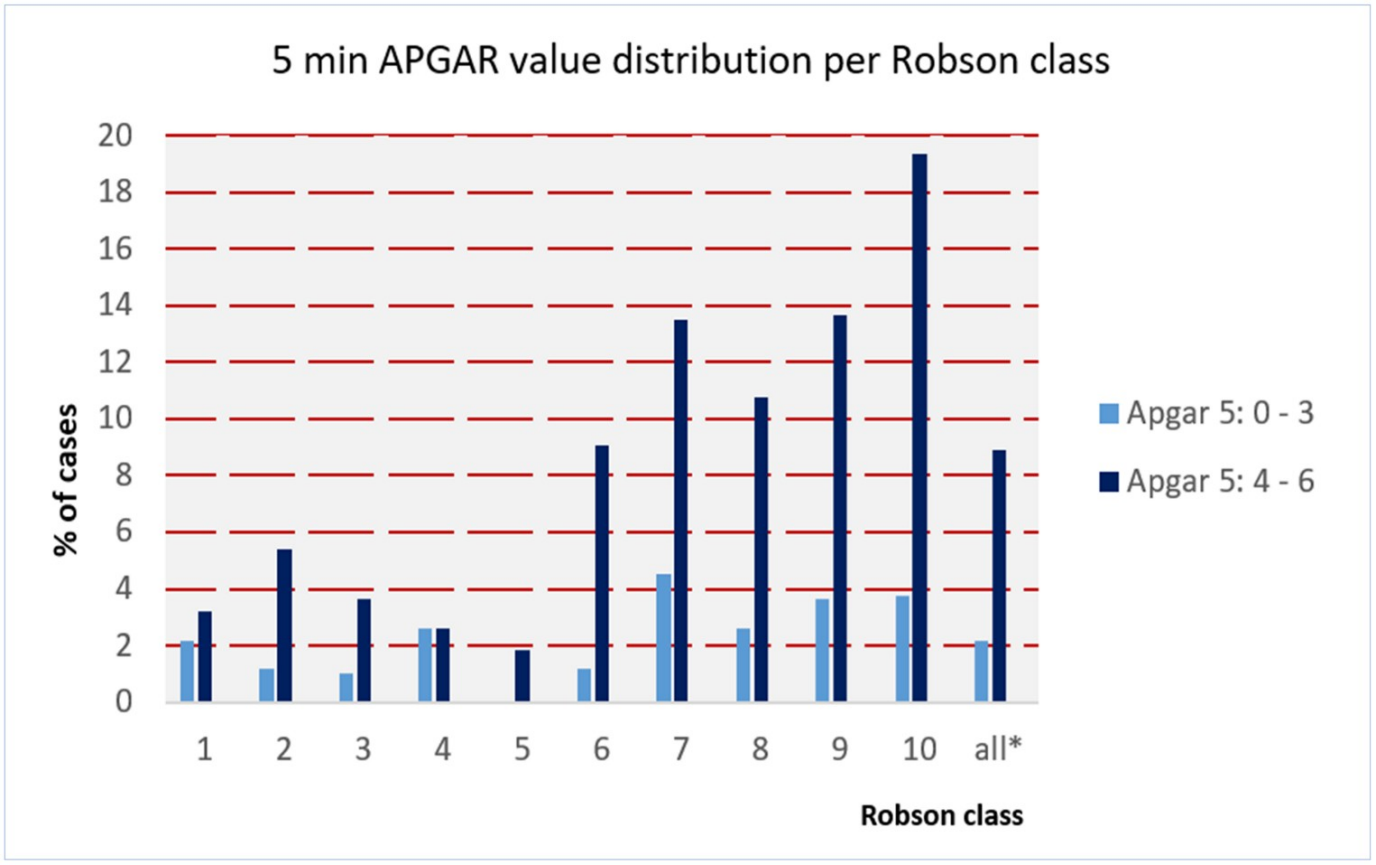
Neonates 5 min APGAR values 0–3 and 4–6 per Robson class % of cases; a 5 min APGAR value > is considered to be normal.

**Fig 4:**
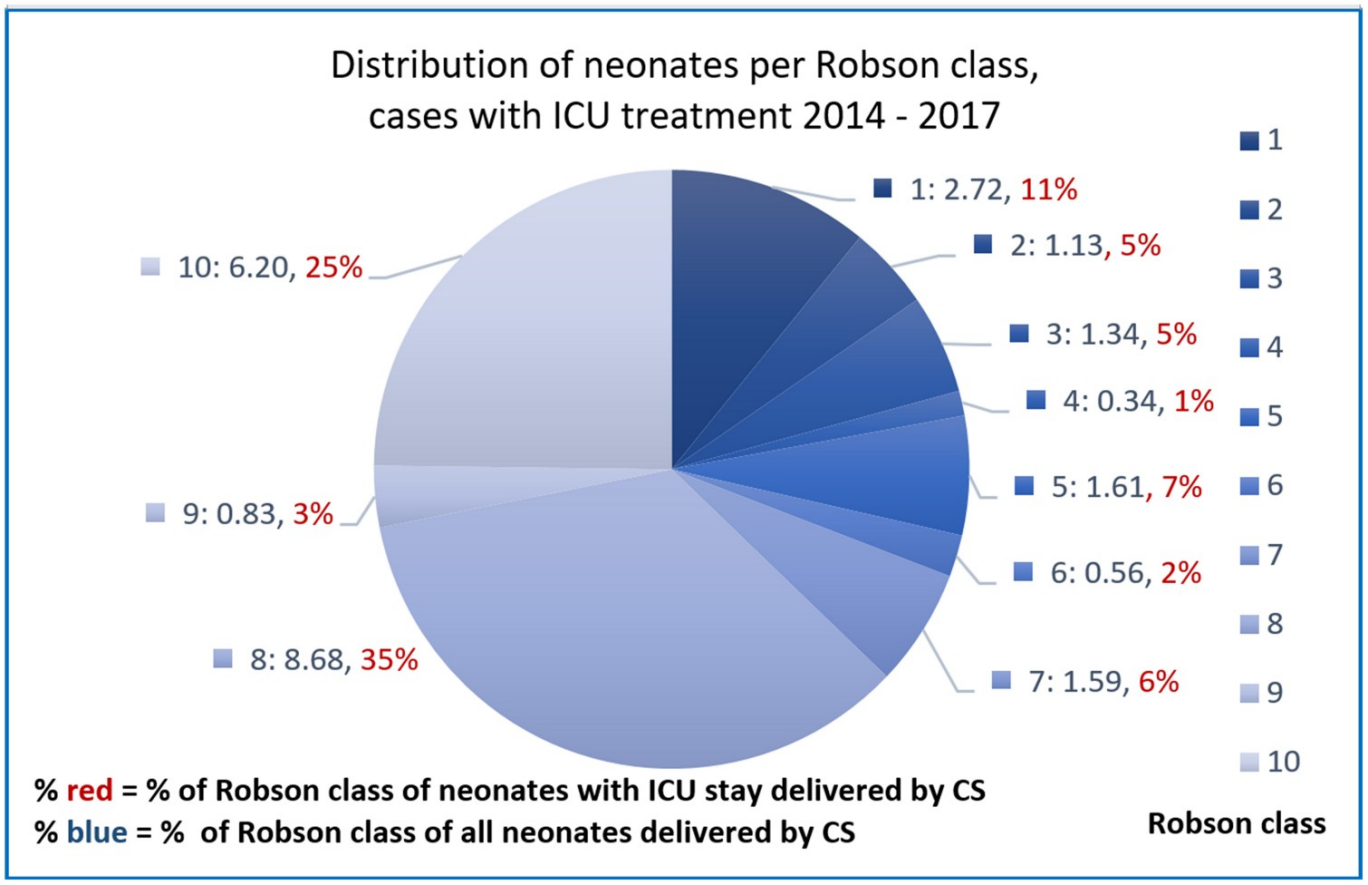
Distribution of Robson classes per newborn cases with intensive care unit (ICU) treatment.

Furthermore, benchmarking cases of mother and child with cephalic on term pregnancies for outcome and complications was made possible by associating outcome information. The distribution of clinical indicators, ICD codes, intensive care unit treatment, APGAR score and DRG type SwissDRG (see Figs 2–4 and S3 Table) could be successfully mapped to Robson classes of cases with CS and the corresponding newborn.

Case related costs could be mapped to the distinct classes and a comparison of the distribution of costs and relevant differences between DRGs and Robson classes could be conducted (see S3 Fig, S4 and S5 Tables, S4 and S5 Figs). The results showed a relevant contribution to cost based grouping only in a few Robson groups (see S6 Table).

**Fig 5:**
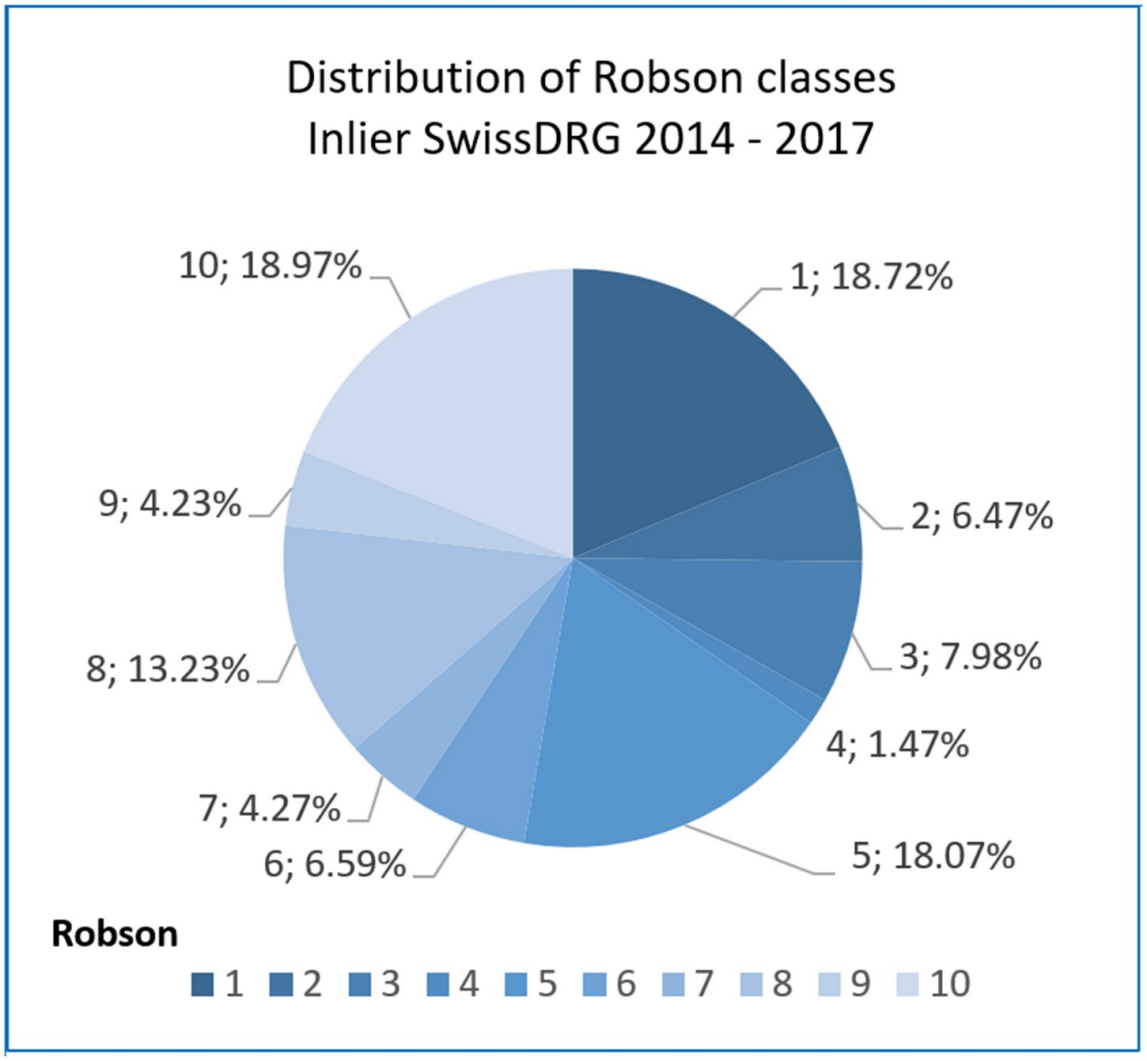
Distribution of Robson classes: Inlier SwissDRG mother cases (batch grouper SwissDRG [30]).

**Fig 6:**
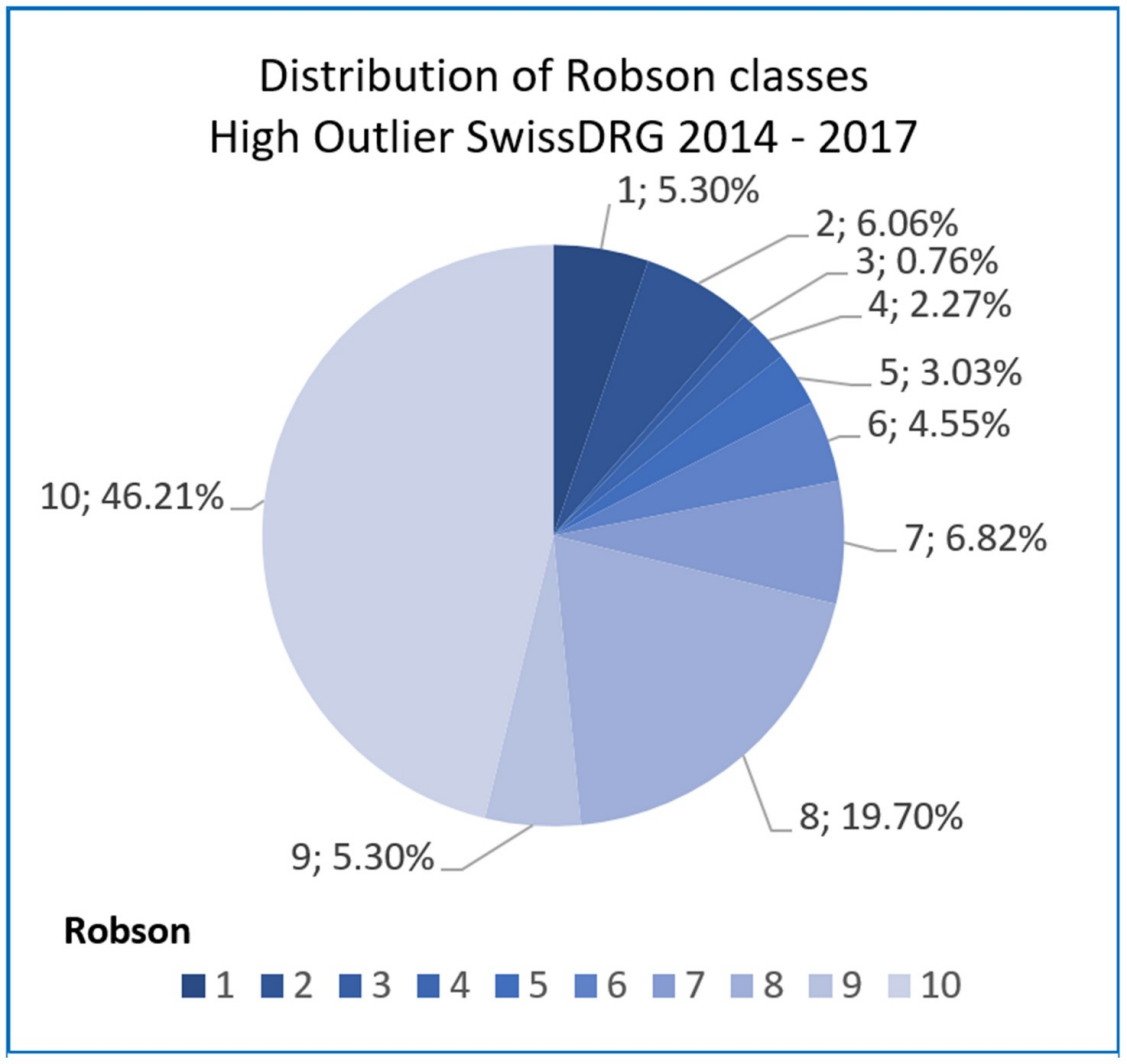
Distribution of Robson classes: High outlier SwissDRG mother cases (batch grouper SwissDRG [30]).

Analyzing the DRG type (length of stay) of mother cases per Robson class it could be demonstrated that the distribution of inlier and high outliers differs in the Robson classes: in class 10, 5, 3 and 1 with inliers distributed to Robson class 10 in 18.97% of cases, class 5 in 18.07%, class 3 in 7.98% and class 1 in 18.72% of the cases respectively, see S7 Table. High outliers were distributed as follows: 46.21% to Robson class 10, 3.03% to class 5, 0.76% to class 3, and 5.03% to class 1 (see Figs 5 and 6).

## 4 Discussion and conclusion

With rising numbers in CS it is essential to improve the data base for benchmarking by implementing valid classifications to work with, especially for elective CS and births at term with cephalic lies. We successfully demonstrated, that a query on routinely collected health data could serve for a complex medical classification. Even with variables from several sources including text mining for missing information it was possible to achieve a complete database for classification (missing values in only 3 cases), a method for benchmarking and monitoring of quality and outcome. With a few Robson groups still showing unexpected results concerning the distribution of cases compared to other studies [33], a thorough validation of the programmed algorithm referring to international standards and the coding rules will be planned for a future study. This must include an analysis of cases with spontaneous delivery. Having conducted these further studies, it will be possible to compare case related costs of the Robson classes to SwissDRGs in order to compare the consistency of the groups. However, as the Robson classification is meant to benchmark quality and outcome it might not be capable of contributing to recovery of costs in DRG systems. Risk-stratification might be conducted using this data set and should be the next step in order to evaluate the Robson criteria and outcome indicators [26,34]. The analysis of outcome data might influence quality monitoring, benchmarking. Applying the method to the group of spontaneous deliveries could be helpful, in order to benchmark caesarean rates. The method could be easily adopted to the data of spontaneous births’ cases by (change of selection criteria).

### 4.1 Strengths

The routinely collected dataset showed a very good quality when referring to completeness and consistency. The connection of laboratory, medical, financial and administrative data on the individual patient’s level with regard to mother and the corresponding child demonstrates a novel approach. The method itself can be universally adapted because an international catalogue of diagnosis codes (ICD) and standardized variables as gestational age are used for the query.

### 4.2 Weaknesses

As national coding rules differ and coding rules in Switzerland underwent changes during the last years, the numbers per Robson class show a limited comparability to those of recent studies extracting the relevant information for classification manually from the patients’ health record. This has to be taken into account when implementing the method using ICD coding information.

### 4.3 Limitations

The verification of the method is based on data and patient groups from one single hospital in the context of Swiss national coding regulations. Moreover, as the study served as a technical proof of concept, only the patient group of CS was analyzed. Therefore, the study is limited to conclusions concerning the distribution of Robson classes within the group of CS cases. No conclusions can be drawn concerning the distribution of CS regarding all births’ modes. Risk-stratification and differences to coding approaches elsewhere have to be elaborated before using this method for international benchmarking.

### 4.4 Conclusion

It is possible to set up an automated method to categorize patient groups according to complex medical classifications based on routinely collected health data. This study might enhance the discussion to adopt the automated classification on routinely collected health data in Switzerland and elsewhere for benchmarking, as outcome variables could be successfully associated to the specific Robson classes and the classification of cases can be conducted efficiently. The method proofed to be capable of applying outcome variables to the complex classification.

## Supporting information

S1 File Classification of caesarean sections, Robson et al., 2002. (PNG)

S1 Table Total count women and corresponding newborns per fiscal year. (DOCX)

S2 Table ICD 10 GM, maternal ICD indicators applicable in 2014–2017. (DOCX)

S3 Table Outcome variables pH, BE, APGAR per Robson class neonates, number of cases. (DOCX)

S4 Table Median and mean case related costs per Robson class mother cases 2014–2017. (DOCX)

S5 Table Log 10 case related costs cases mother per Robson class and year, variation coefficient, median, mean and standard deviation. (DOCX)

S6 Table Distribution of log 10 case related costs of mother cases per Robson class 2014–2017. (DOCX)

S7 Table Number of lier types per Robson class. (DOCX)

S1 Fig Flowchart of selection process. (TIF)

S2 Fig Robson data flow for algorithm; Roger mathis IT Inselspital. (TIF)

S3 Fig Log 10 costs. (TIF)

S4 Fig Distribution of log 10 case related costs of mother cases per DRG 2014–2017. (TIF)

S5 Fig Pairwise t test Robson classes mother cases per year. (TIF)

## References

[1] Betrán AP, Ye J, Moller AB, Zhang J, Gülmezoglu AM, Torloni MR. The increasing trend in caesarean section rates: Global, regional and national estimates: 1990–2014. *PLoS One*. 2016;11(2). 10.1371/journal.pone.0148343 26849801 PMC4743929

[2] WHO | WHO statement on caesarean section rates. WHO. 2019.

[3] EUROPEAN PERINATAL HEALTH REPORT. www.europeristat.com. Accessed October 9, 2019.

[4] Torloni MR, Betran AP, Souza JP, et al Classifications for cesarean section: A systematic review. *PLoS One*. 2011;6(1). 10.1371/journal.pone.0014566 21283801 PMC3024323

[5] Vogel JP, Betrán AP, Vindevoghel N, et al Use of the robson classification to assess caesarean section trends in 21 countries: A secondary analysis of two WHO multicountry surveys. *Lancet Glob Heal*. 2015;3(5):e260–e270. 10.1016/S2214-109X(15)70094-X 25866355

[6] Slavin V, Fenwick J. Use of a Classification Tool to Determine Groups of Women That Contribute to the Cesarean Section Rate: Establishing a Baseline for Clinical Decision Making and Quality Improvement. *Int J Childbirth*. 2012;2 10.1891/2156-5287.2.3.187 26594592 PMC4652937

[7] Farine D, Shepherd D. Classification of caesarean sections in Canada: the modified robson criteria. *J Obs Gynaecol Can*. 2012;34. 25162083

[8] Robson M, Hartigan L, Murphy M. Methods of achieving and maintaining an appropriate caesarean section rate. *Best Pract Res Clin Obstet Gynaecol*. 2013;27(2):297–308. 10.1016/j.bpobgyn.2012.09.004 23127896

[9] Robson M. The ten group classification system (TGCS)-a common starting point for more detailed analysis. *BJOG An Int J Obstet Gynaecol*. 2015;122(5):701 10.1111/1471-0528.13267 25600521

[10] Melman S, Schoorel ECN, De Boer K, et al Development and measurement of guidelines-based quality indicators of caesarean section care in the Netherlands: A RAND-modified delphi procedure and retrospective medical chart review. *PLoS One*. 2016;11(1). 10.1371/journal.pone.0145771 26783742 PMC4718610

[11] Leitlinien SGGG-DGGG-OeGGG—SGGG. https://www.sggg.ch/fachthemen/leitlinien-sggg-dggg-oeggg/. Accessed October 9, 2019.

[12] Betrán AP, Vindevoghel N, Souza JP, Gülmezoglu AM, Torloni MR. A systematic review of the Robson classification for caesarean section: What works, doesn’t work and how to improve it. *PLoS One*. 2014;9(6). 10.1371/journal.pone.0097769 24892928 PMC4043665

[13] Robson MS. Classification of caesarean sections. *Fetal Matern Med Rev*. 2001;12(1):23–39. 10.1017/S0965539501000122

[14] WHO | Robson Classification: Implementation Manual. WHO. 2019.

[15] Brennan DJ, Murphy M, Robson MS, O’Herlihy C. The singleton, cephalic, nulliparous woman after 36 weeks of gestation: Contribution to overall cesarean delivery rates. *Obstet Gynecol*. 2011;117(2):273–279. 10.1097/AOG.0b013e318204521a 21252739

[16] Marshall NE, Fu R, Guise JM. Impact of multiple cesarean deliveries on maternal morbidity: A systematic review. In: *American Journal of Obstetrics and Gynecology*. Vol 205 Mosby Inc.; 2011:262.e1–262.e8. 10.1016/j.ajog.2011.06.035 22071057

[17] Warren J. Big Data: Principles and Best Practices Nathan Marz. http://nathanmarz.com/about/. Accessed October 10, 2019.

[18] Sharabiani MTA, Aylin P, Bottle A. Systematic review of comorbidity indices for administrative data. *Med Care*. 2012;50(12):1109–1118. 10.1097/MLR.0b013e31825f64d0 22929993

[19] Über die Klinik—Universitätsklinik für Frauenheilkunde. http://www.frauenheilkunde.insel.ch/de/ueber-die-klinik/. Accessed October 10, 2019.

[20] Joseph KS, Kramer MS. The fetuses-at-risk approach: survival analysis from a fetal perspective. *Acta Obstet Gynecol Scand*. 2018;97(4):454–465. 10.1111/aogs.13194 28742216 PMC5887948

[21] Leone A, Ersfeld P, Adams M, Meyer Schiffer P, Bucher HU, Arlettaz R. Neonatal morbidity in singleton late preterm infants compared with full-term infants. *Acta Paediatr Int J Paediatr*. 2012;101(1). 10.1111/j.1651-2227.2011.02459.x 21895764

[22] Andersson CB, Flems C, Kesmodel US. The Danish national quality database for births. *Clin Epidemiol*. 2016;8:595–599. 10.2147/CLEP.S99492 27822105 PMC5094609

[23] Yurkovich M, Avina-Zubieta JA, Thomas J, Gorenchtein M, Lacaille D. A systematic review identifies valid comorbidity indices derived from administrative health data. *J Clin Epidemiol*. 2015;68(1):3–14. 10.1016/j.jclinepi.2014.09.010 25441702

[24] Stausberg J, Hagn S. New morbidity and comorbidity scores based on the structure of the ICD-10. *PLoS One*. 2015;10(12). 10.1371/journal.pone.0143365 26656501 PMC4677989

[25] Kesmodel US, Jã̃lving LR. Measuring and improving quality in obstetrics—The implementation of national indicators in Denmark. *Acta Obstet Gynecol Scand*. 2011;90(4):295–304. 10.1111/j.1600-0412.2011.01078.x 21306336

[26] Campbell SM, Braspenning J, Hutchinson A, Marshall MN. Research methods used in developing and applying quality indicators in primary care. *BMJ*. 2003;326(7393):816–819. 10.1136/bmj.326.7393.816 12689983 PMC1125721

[27] DIMDI—ICD-10-GM Version 2016. https://www.dimdi.de/static/de/klassifikationen/icd/icd-10-gm/kode-suche/htmlgm2016/. Accessed October 10, 2019.

[28] Instrumente zur medizinischen Kodierung. https://www.bfs.admin.ch/bfs/de/home/statistiken/gesundheit/nomenklaturen/medkk/instrumente-medizinische-kodierung.html. Accessed October 10, 2019.

[29] Committee Opinion No. 644: The Apgar Score. *Obstet Gynecol*. 2015;126(4):e52–e55. 10.1097/AOG.0000000000001108 26393460

[30] Swiss DRG AG. SwissDRG AG. http://www.swissdrg.org/de/index.asp?navid=0. Published 2016.

[31] Besson P. Zertifizierungsverfahren REKOLE^®^ –H+ Die Spitäler der Schweiz. H+ Die Spitäler der Schweiz. https://www.hplus.ch/de/rechnungswesen/zertifizierungsverfahren-rekole/. Published 2018. Accessed June 12, 2019.

[32] Handbuch REKOLE^®^ –H+ Die Spitäler der Schweiz. https://www.hplus.ch/de/rechnungswesen/handbuch-rekole/. Accessed October 10, 2019.

[33] Hehir MP, Ananth C V., Siddiq Z, Flood K, Friedman AM, D’Alton ME. Cesarean delivery in the United States 2005 through 2014: a population-based analysis using the Robson 10-Group Classification System. *Am J Obstet Gynecol*. 2018;219(1):105.e1–105.e11. 10.1016/j.ajog.2018.04.012 29655965

[34] Colais P, Fantini MP, Fusco D, et al Risk adjustment models for interhospital comparison of CS rates using Robson’s ten group classification system and other socio-demographic and clinical variables. *BMC Pregnancy Childbirth*. 2012;12 10.1186/1471-2393-12-54 22720844 PMC3570355

