# Supplementary figures and images for: The Robson classification for caesarean section—A proposed method based on routinely collected health data
Source: PLoS One. 2020 Nov 30;15(11):e0242736. doi: 10.1371/journal.pone.0242736 (PMC7703923; doi:10.1371/journal.pone.0242736)

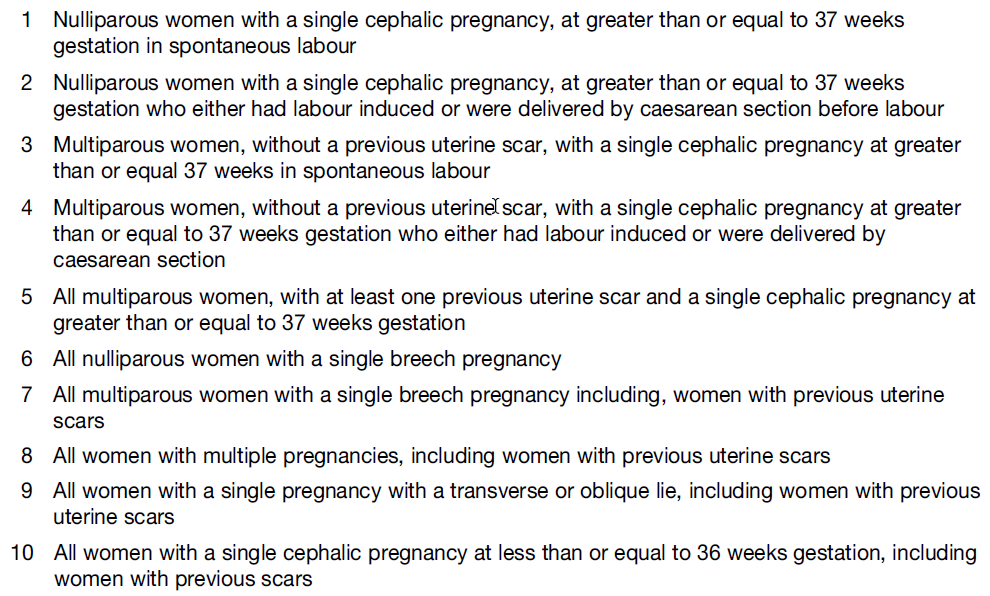

Supplement: S1 File — (PNG) [file pone.0242736.s001.png]

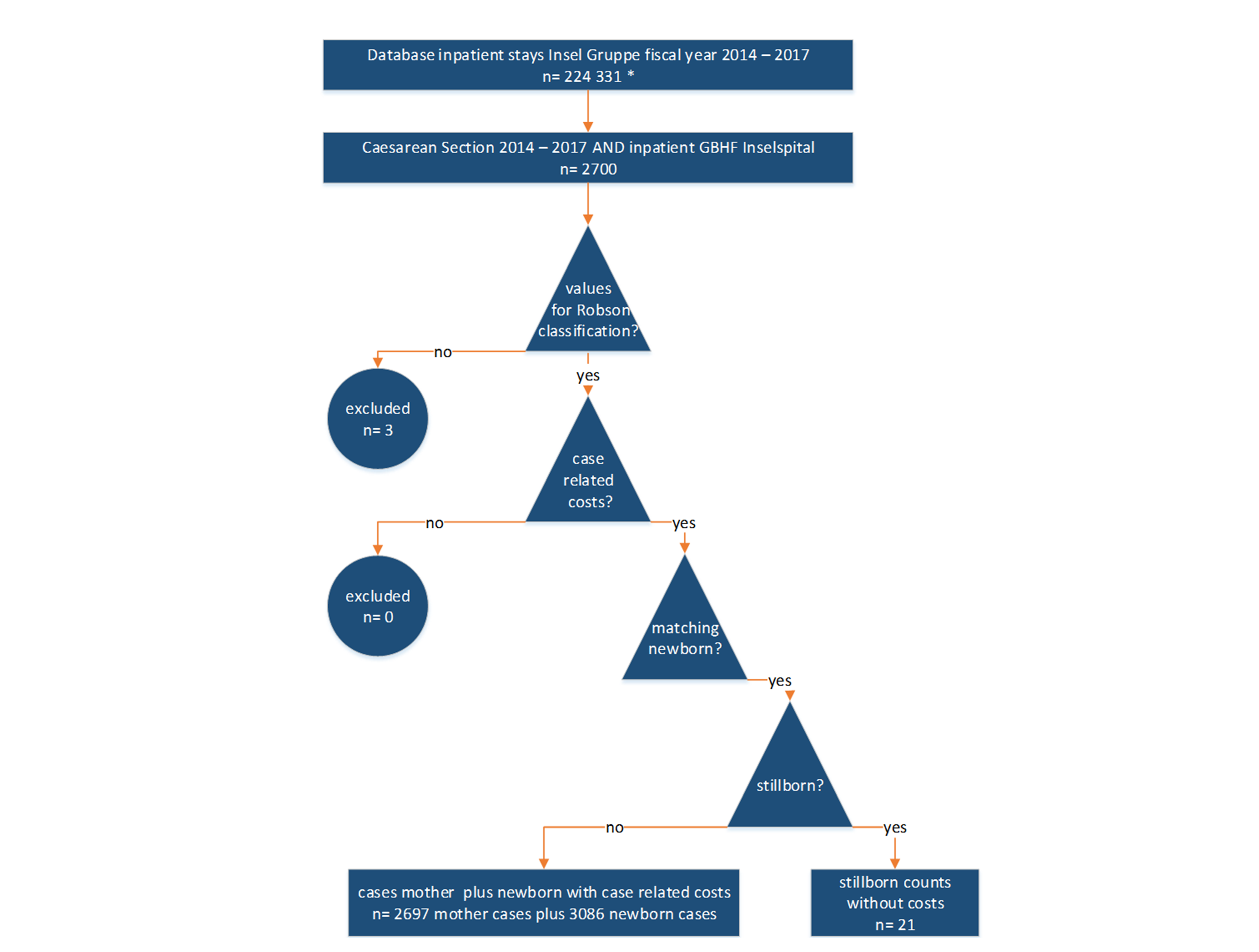

Supplement: S1 Fig — (TIF) [file pone.0242736.s009.tif]

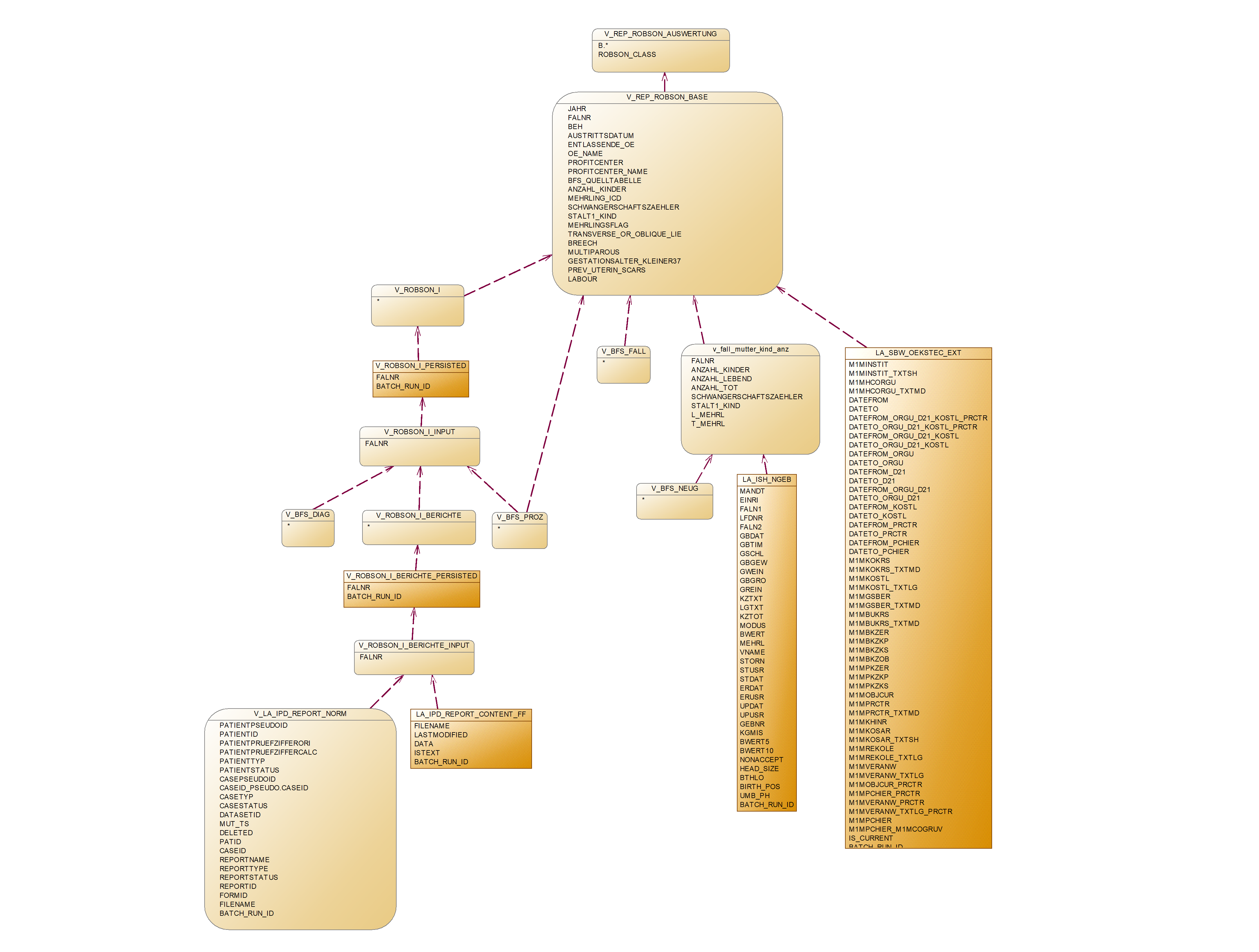

Supplement: S2 Fig — (TIF) [file pone.0242736.s010.tif]

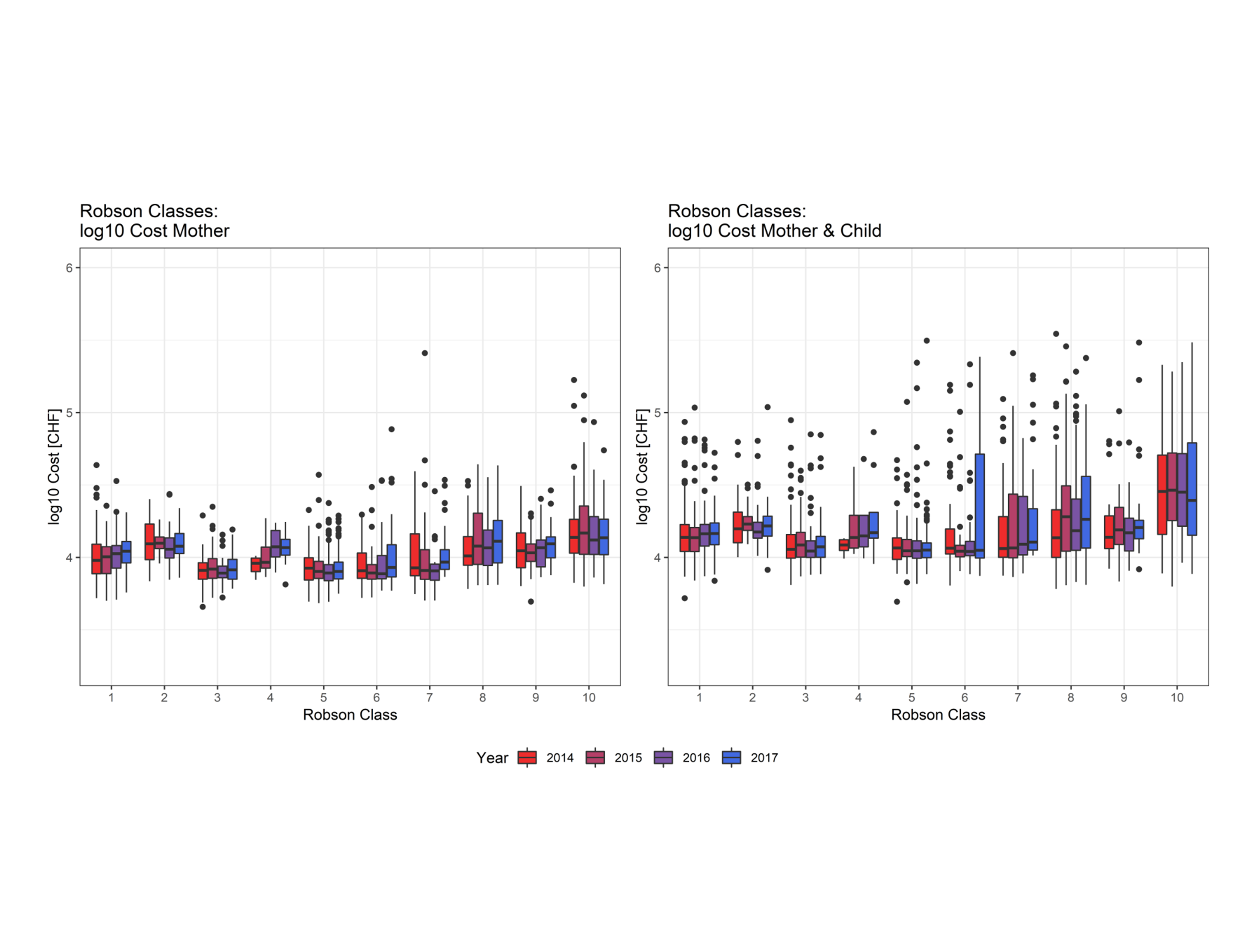

Supplement: S3 Fig — (TIF) [file pone.0242736.s011.tif]

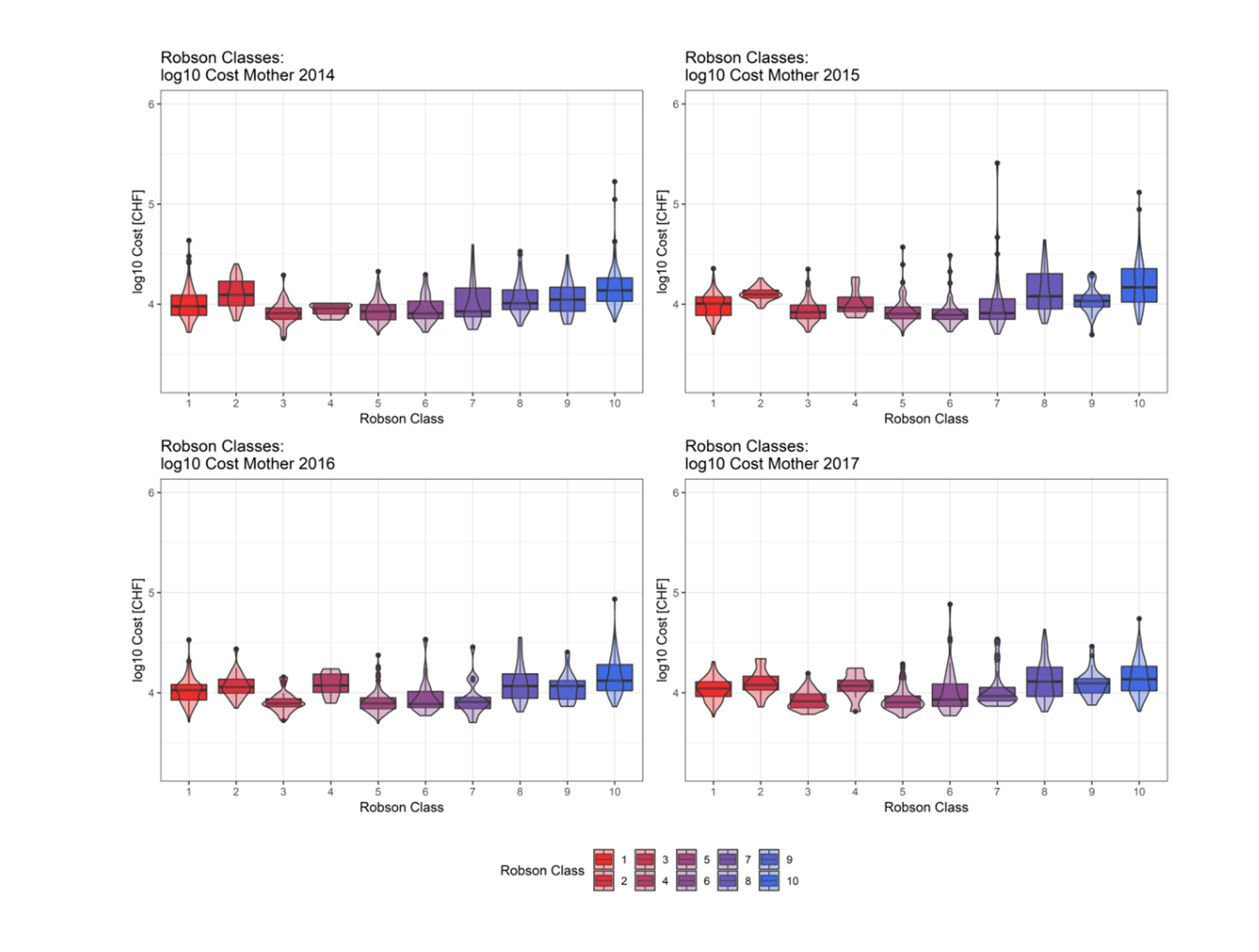

Supplement: S4 Fig — (TIF) [file pone.0242736.s012.tif]

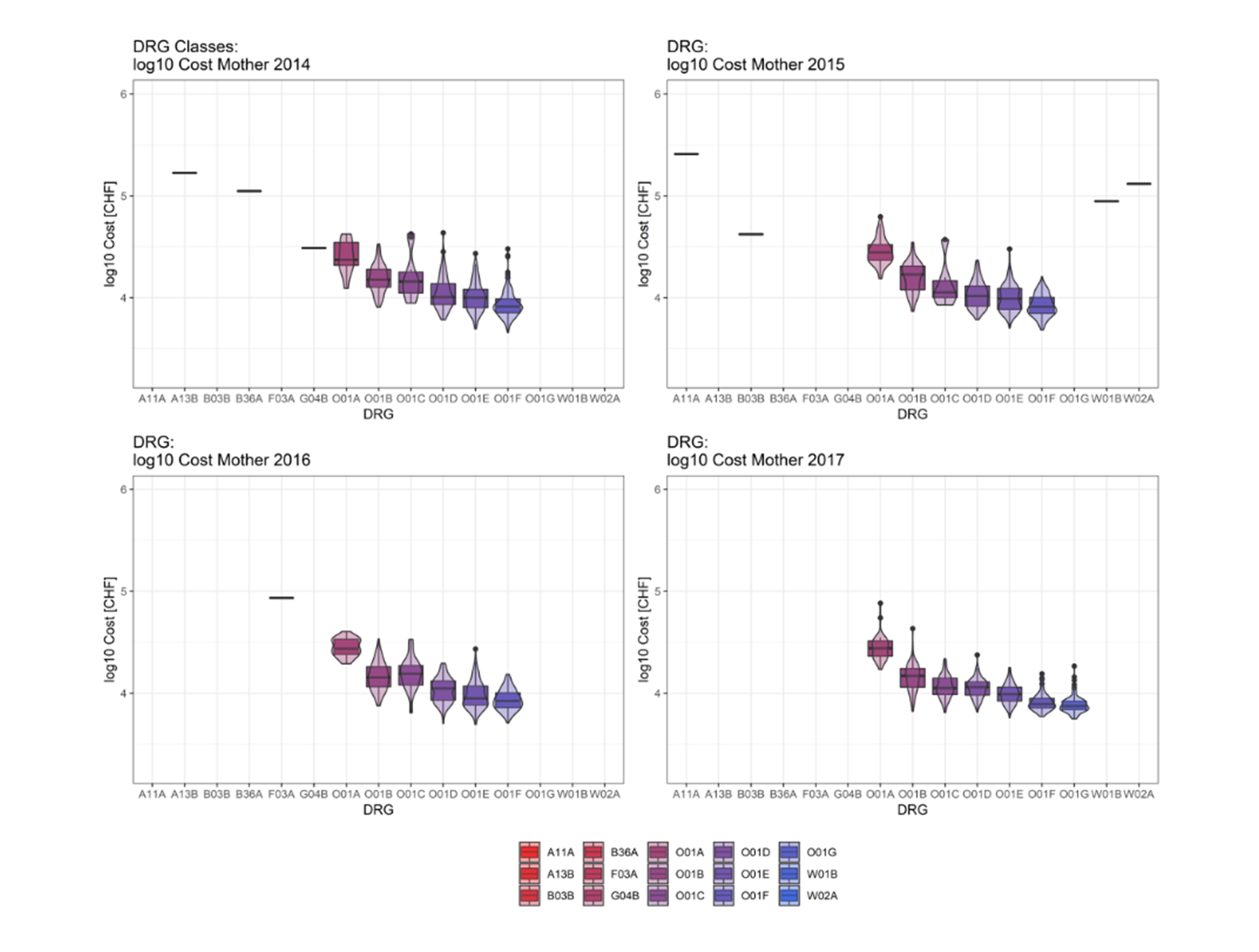

Supplement: S5 Fig — (TIF) [file pone.0242736.s013.tif]
