# Supplementary material for: The Robson classification for caesarean section—A proposed method based on routinely collected health data
Source: PLoS One. 2020 Nov 30;15(11):e0242736. doi: 10.1371/journal.pone.0242736 (PMC7703923; doi:10.1371/journal.pone.0242736)
Supplement: S1 Table — (DOCX) [file pone.0242736.s002.docx]

S1 Table. Total count women and corresponding newborns per fiscal year

| **fiscal year** | **inpatient stays** | | |
| --- | --- | --- | --- |
|  | **count cases delivery by CS** | **count cases matching newborn** | **count cases matching stillborn** |
| **2014** | 647 | 735 | 1 |
| **2015** | 681 | 802 | 12* |
| **2016** | 661 | 779 | 4 |
| **2017** | 711 | 791 | 4 |

*changes in the administrative process concerning late abortions
