# Supplementary material for: The Robson classification for caesarean section—A proposed method based on routinely collected health data
Source: PLoS One. 2020 Nov 30;15(11):e0242736. doi: 10.1371/journal.pone.0242736 (PMC7703923; doi:10.1371/journal.pone.0242736)
Supplement: S2 Table — (DOCX) [file pone.0242736.s003.docx]

S2 Table. ICD 10 GM, maternal ICD indicators applicable in 2014 – 2017

| **ICD code** | **text** |
| --- | --- |
| O43ff | Placental complications |
| O72ff | Bleeding complications |
| O75.1 | Shock post partum |
| O85 | Puerperalfieber |
| O86.0 | Infektion der Wunde nach operativem geburtshilflichem Eingriff |
| O86.1 | Sonstige Infektion des Genitaltraktes nach Entbindung |
| O86.2 | Infektion des Harntraktes nach Entbindung |
| O86.3 | Sonstige Infektionen des Urogenitaltraktes nach Entbindung |
| O88ff | Thrombosis, embolia |
| T81.4 | Infektion nach einem Eingriff, anderenorts nicht klassifiziert |
| A41.0 | Sepsis: Staph aureus |
| A41.1 | Sepsis: Staph sonstige |
| A41.2 | Sepsis: Staph nnb |
| A41.3 | Sepsis: Haemophilus influenza |
| A41.4 | Sepsis: Anaerobier |
| A41.51 | Sepsis: E. coli |
| A41.52 | Sepsis: Pseudomonas |
| A41.58 | Sepsis: gram neg sonst |
| A41.8 | sonst näher bezeichnete Sepsis |
| A41.9 | Sepsis nicht näher bezeichnet |
| A40.0 | Sepsis: Strepto A |
| A40.1 | Sepsis: Strepto B |
| A40.2 | Sepsis: Strepto D |
| A40.3 | Sepsis: Strept pneumonia |
| A40.8 | Sepsis: sonst Strepto |
| A40.9 | Sepsis: Epsis. strepto nnb |
| A39.2 | akute Meningokokkensepsis |
| A39.4 | Meningokokkensepsis nnb |
| A02.1 | Salmonellen sepsis |
| A32.7 | Listeriensepsis |
| A39.1 | Waterhouse-Friderichsen-Syndrom |
| A42.7 | Aktinomykotische Sepsis |
| B37.7 | Candidasepsis |
| R57.2 | Septischer Schock |
| T81.0 | Bleeding, intraoperative |
| T81.1 | Shock, perioperative |
| T81.2 | Infection, postoperative |
