# Supplementary material for: The Robson classification for caesarean section—A proposed method based on routinely collected health data
Source: PLoS One. 2020 Nov 30;15(11):e0242736. doi: 10.1371/journal.pone.0242736 (PMC7703923; doi:10.1371/journal.pone.0242736)
Supplement: S3 Table — (DOCX) [file pone.0242736.s004.docx]

|  | Robson class | | | | | | | | | |
| --- | --- | --- | --- | --- | --- | --- | --- | --- | --- | --- |
|  | 1 | 2 | 3 | 4 | 5 | 6 | 7 | 8 | 9 | 10 |
| outcome variable |  |  |  |  |  |  |  |  |  |  |
| pH < 7.00 | 5 | 2 | 3 | 1 | 3 | 0 | 2 | 8 | 1 | 3 |
| pH 7.00-7.09 | 6 | 10 | 6 | 0 | 3 | 2 | 1 | 3 | 1 | 7 |
| BE - 6 to -12 | 23 | 12 | 10 | 0 | 12 | 3 | 1 | 10 | 1 | 10 |
| BE under -12 | 2 | 1 | 3 | 1 | 0 | 0 | 1 | 4 | 1 | 3 |
| Apgar 5: 0 - 3 | 10 | 2 | 2 | 1 | 0 | 2 | 5 | 18 | 4 | 19 |
| Apgar 5: 4 - 6 | 15 | 9 | 7 | 1 | 8 | 15 | 15 | 75 | 15 | 98 |
| Apgar 5: 7 - 10 | 442 | 155 | 184 | 36 | 432 | 149 | 91 | 605 | 91 | 390 |
