# Supplementary material for: The Robson classification for caesarean section—A proposed method based on routinely collected health data
Source: PLoS One. 2020 Nov 30;15(11):e0242736. doi: 10.1371/journal.pone.0242736 (PMC7703923; doi:10.1371/journal.pone.0242736)
Supplement: S4 Table — (DOCX) [file pone.0242736.s005.docx]

S4 Table. Median and mean case related costs per Robson class mother cases 2014 - 2017

| **median** | **year** | | | |
| --- | --- | --- | --- | --- |
| **Robson class** | **2014** | **2015** | **2016** | **2017** |
| **1** | 9527 | 9705 | 9847 | 10387 |
| **2** | 12393 | 10200 | 11699 | 10395 |
| **3** | 8134 | 9844 | 9756 | 9525 |
| **4** | 9104 | 10869 | 8496 | 8990 |
| **5** | 8432 | 9995 | 9198 | 9561 |
| **6** | 8096 | 9141 | 10053 | 10563 |
| **7** | 8463 | 9281 | 10600 | 8664 |
| **8** | 10229 | 10868 | 9833 | 10274 |
| **9** | 11111 | 8981 | 8246 | 9551 |
| **10** | 13739 | 10056 | 10718 | 10303 |
|  |  |  |  |  |
| **mean** | **year** | | | |
| **Robson class** | **2014** | **2015** | **2016** | **2017** |
| **1** | 10925 | 12644 | 11640 | 12154 |
| **2** | 13462 | 12013 | 13442 | 11244 |
| **3** | 8386 | 11806 | 11075 | 11221 |
| **4** | 8854 | 12128 | 9206 | 10879 |
| **5** | 8903 | 12927 | 10887 | 13085 |
| **6** | 9315 | 11360 | 14917 | 11903 |
| **7** | 11425 | 11192 | 11078 | 8829 |
| **8** | 12396 | 12302 | 11850 | 11589 |
| **9** | 12579 | 9799 | 9674 | 9849 |
| **10** | 17142 | 11677 | 13499 | 13219 |
