# Supplementary material for: The Robson classification for caesarean section—A proposed method based on routinely collected health data
Source: PLoS One. 2020 Nov 30;15(11):e0242736. doi: 10.1371/journal.pone.0242736 (PMC7703923; doi:10.1371/journal.pone.0242736)
Supplement: S5 Table — (DOCX) [file pone.0242736.s006.docx]

| **var** | **year** | | | |
| --- | --- | --- | --- | --- |
| **Robson class** | **2014** | **2015** | **2016** | **2017** |
| **1** | 0.0265925 | 0.043797308 | 0.031758683 | 0.029002975 |
| **2** | 0.022855443 | 0.029826878 | 0.041506025 | 0.018543528 |
| **3** | 0.012682026 | 0.043366842 | 0.023467932 | 0.028028181 |
| **4** | 0.003800435 | 0.028710192 | 0.013010929 | 0.028388219 |
| **5** | 0.013241247 | 0.039099107 | 0.030202415 | 0.047818554 |
| **6** | 0.018992188 | 0.032272362 | 0.057150862 | 0.035288553 |
| **7** | 0.045286893 | 0.029636867 | 0.023818004 | 0.006129628 |
| **8** | 0.027461598 | 0.031296824 | 0.030600463 | 0.032645378 |
| **9** | 0.030249563 | 0.017341001 | 0.021847908 | 0.010670559 |
| **10** | 0.041446128 | 0.028249128 | 0.040002009 | 0.044892936 |
|  |  |  |  |  |
| **median** | **year** | | | |
| **Robson class** | **2014** | **2015** | **2016** | **2017** |
| **1** | 3.978956165 | 3.98697012 | 3.993276344 | 4.016469044 |
| **2** | 4.093163957 | 4.008575112 | 4.068148741 | 4.016597061 |
| **3** | 3.910304168 | 3.993171605 | 3.989191141 | 3.978837231 |
| **4** | 3.959232249 | 4.036107701 | 3.929214504 | 3.953759692 |
| **5** | 3.925925711 | 3.999782798 | 3.963693405 | 3.980480472 |
| **6** | 3.908266441 | 3.960989551 | 4.002295663 | 4.02378728 |
| **7** | 3.927524341 | 3.966950913 | 4.025305865 | 3.937718444 |
| **8** | 4.009803788 | 4.036128049 | 3.992686039 | 4.011699237 |
| **9** | 4.045691631 | 3.953324696 | 3.916236276 | 3.979894035 |
| **10** | 4.137938683 | 4.002425265 | 4.030113753 | 4.0129637 |
|  |  |  |  |  |
| **mean** | **year** | | | |
| **Robson class** | **2014** | **2015** | **2016** | **2017** |
| **1** | 4.003703746 | 4.021839753 | 4.025203623 | 4.049209313 |
| **2** | 4.103537339 | 4.044658961 | 4.077416098 | 4.030724984 |
| **3** | 3.908479718 | 4.014853571 | 4.01552241 | 4.016741539 |
| **4** | 3.943325793 | 4.053808594 | 3.951058752 | 4.007520341 |
| **5** | 3.933286134 | 4.051125348 | 3.998269531 | 4.032584539 |
| **6** | 3.945967373 | 4.011802141 | 4.07265172 | 4.030733634 |
| **7** | 3.99731796 | 4.013200464 | 4.017270255 | 3.939020319 |
| **8** | 4.05892569 | 4.05102235 | 4.0349329 | 4.024505114 |
| **9** | 4.064289914 | 3.970821353 | 3.958528022 | 3.981695399 |
| **10** | 4.165516479 | 4.03086274 | 4.059475728 | 4.054844636 |
|  |  |  |  |  |
| **SD** | **year** | | | |
| **Robson class** | **2014** | **2015** | **2016** | **2017** |
| **1** | 0.163072069 | 0.209278063 | 0.178209661 | 0.170302598 |
| **2** | 0.151180167 | 0.172704598 | 0.203730275 | 0.136174624 |
| **3** | 0.112614503 | 0.20824707 | 0.153192467 | 0.167416191 |
| **4** | 0.061647671 | 0.169440821 | 0.114065458 | 0.168488038 |
| **5** | 0.11507062 | 0.19773494 | 0.17378842 | 0.218674538 |
| **6** | 0.137812146 | 0.179645101 | 0.239062464 | 0.187852478 |
| **7** | 0.212807173 | 0.172153614 | 0.154330827 | 0.078291943 |
| **8** | 0.165715413 | 0.176909084 | 0.17492988 | 0.180680319 |
| **9** | 0.173924015 | 0.131685234 | 0.147810378 | 0.103298397 |
| **10** | 0.203583222 | 0.168074769 | 0.200005023 | 0.211879531 |
