# Supplementary material for: The Robson classification for caesarean section—A proposed method based on routinely collected health data
Source: PLoS One. 2020 Nov 30;15(11):e0242736. doi: 10.1371/journal.pone.0242736 (PMC7703923; doi:10.1371/journal.pone.0242736)
Supplement: S6 Table — (DOCX) [file pone.0242736.s007.docx]

| **Robson Class** |  |  |  |  |  |  |  |  |  |
| --- | --- | --- | --- | --- | --- | --- | --- | --- | --- |
| **2017** |  |  |  |  |  |  |  |  |  |
|  | **p- values** | | | | | | | | |
|  | **1** | **2** | **3** | **4** | **5** | **6** | **7** | **8** | **9** |
| **2** | NA | NA | NA | NA | NA | NA | NA | NA | NA |
| **3** | 4.96E-11 | 1.09E-05 | NA | NA | NA | NA | NA | NA | NA |
| **4** | NA | NA | NA | NA | NA | NA | NA | NA | NA |
| **5** | 8.36E-14 | 1.06E-05 | NA | NA | NA | NA | NA | NA | NA |
| **6** | NA | NA | NA | NA | NA | NA | NA | NA | NA |
| **7** | NA | NA | NA | NA | NA | NA | NA | NA | NA |
| **8** | 0.00303948 | NA | 1.00E-10 | NA | 4.96E-11 | NA | NA | NA | NA |
| **9** | NA | NA | 7.68E-07 | NA | 6.44E-07 | NA | NA | NA | NA |
| **10** | 2.08E-08 | NA | 7.22E-22 | NA | 8.33E-24 | 0.00422881 | NA | NA | NA |
|  |  |  |  |  |  |  |  |  |  |
| **2016** |  |  |  |  |  |  |  |  |  |
|  | **p- values** | | | | | | | | |
|  | **1** | **2** | **3** | **4** | **5** | **6** | **7** | **8** | **9** |
| **2** | NA | NA | NA | NA | NA | NA | NA | NA | NA |
| **3** | 5.35E-06 | 7.85E-10 | NA | NA | NA | NA | NA | NA | NA |
| **4** | NA | NA | 0.00405757 | NA | NA | NA | NA | NA | NA |
| **5** | 3.45E-09 | 8.08E-14 | NA | 0.00465828 | NA | NA | NA | NA | NA |
| **6** | NA | 0.00297113 | NA | NA | NA | NA | NA | NA | NA |
| **7** | NA | NA | NA | NA | NA | NA | NA | NA | NA |
| **8** | 0.00342288 | NA | 6.02E-10 | NA | 2.58E-12 | 0.00074847 | 0.00411458 | NA | NA |
| **9** | NA | NA | 0.00044826 | NA | 0.00041011 | NA | NA | NA | NA |
| **10** | 1.62E-09 | 0.00036691 | 4.98E-17 | NA | 2.45E-23 | 4.64E-07 | 0.00010902 | NA | 0.00789019 |
|  |  |  |  |  |  |  |  |  |  |
| **2015** |  |  |  |  |  |  |  |  |  |
|  | **p- values** | | | | | | | | |
|  | **1** | **2** | **3** | **4** | **5** | **6** | **7** | **8** | **9** |
| **2** | 1.27E-10 | NA | NA | NA | NA | NA | NA | NA | NA |
| **3** | NA | 6.89E-12 | NA | NA | NA | NA | NA | NA | NA |
| **4** | NA | NA | NA | NA | NA | NA | NA | NA | NA |
| **5** | 0.00287197 | 5.51E-18 | NA | NA | NA | NA | NA | NA | NA |
| **6** | NA | 8.43E-07 | NA | NA | NA | NA | NA | NA | NA |
| **7** | NA | NA | NA | NA | NA | NA | NA | NA | NA |
| **8** | 9.19E-08 | NA | 1.27E-10 | NA | 1.36E-12 | 1.93E-07 | NA | NA | NA |
| **9** | NA | NA | 0.00555272 | NA | 0.00248532 | NA | NA | NA | NA |
| **10** | 4.66E-14 | 0.00014742 | 1.51E-16 | 0.00358024 | 2.47E-19 | 3.12E-11 | NA | NA | 1.98E-05 |
|  |  |  |  |  |  |  |  |  |  |
| **2014** |  |  |  |  |  |  |  |  |  |
|  | **p- values** | | | | | | | | |
|  | **1** | **2** | **3** | **4** | **5** | **6** | **7** | **8** | **9** |
| **2** | 0.00283216 | NA | NA | NA | NA | NA | NA | NA | NA |
| **3** | 0.00022015 | 1.66E-07 | NA | NA | NA | NA | NA | NA | NA |
| **4** | NA | 9.30E-05 | NA | NA | NA | NA | NA | NA | NA |
| **5** | 0.00114243 | 8.04E-07 | NA | NA | NA | NA | NA | NA | NA |
| **6** | NA | 1.93E-05 | NA | NA | NA | NA | NA | NA | NA |
| **7** | NA | NA | NA | NA | NA | NA | NA | NA | NA |
| **8** | NA | NA | 3.19E-07 | 0.00098655 | 1.34E-06 | 0.00023034 | NA | NA | NA |
| **9** | NA | NA | 0.00064638 | 0.00949606 | 0.00243883 | 0.00911982 | NA | NA | NA |
| **10** | 4.86E-10 | NA | 3.65E-18 | 1.87E-07 | 1.86E-20 | 5.72E-13 | 0.00098655 | 0.00024296 | NA |
