# Supplementary material for: The Robson classification for caesarean section—A proposed method based on routinely collected health data
Source: PLoS One. 2020 Nov 30;15(11):e0242736. doi: 10.1371/journal.pone.0242736 (PMC7703923; doi:10.1371/journal.pone.0242736)
Supplement: S7 Table — (DOCX) [file pone.0242736.s008.docx]

|  |  |  |
| --- | --- | --- |
| Robson class | Inlier | High Outlier |
| 1 | 460 | 7 |
| 2 | 159 | 8 |
| 3 | 196 | 1 |
| 4 | 36 | 3 |
| 5 | 444 | 4 |
| 6 | 162 | 6 |
| 7 | 105 | 9 |
| 8 | 325 | 26 |
| 9 | 104 | 7 |
| 10 | 466 | 61 |
